# Giardiasis: Serum antibodies and coproantigens in brown rats (*Rattus norvegicus*) from Grenada, West Indies

**DOI:** 10.14202/vetworld.2018.293-296

**Published:** 2018-03-12

**Authors:** Keshaw Tiwari, Camille Coomansingh Springer, Alfred Chikweto, Josephine Tang, Yvette Sepulveda, Amanda Leigh Smith, Nia Rametta, Ravindra Nath Sharma

**Affiliations:** Department of Pathobiology, School of Veterinary Medicine, St. George’s University, Grenada, West Indies

**Keywords:** antibody, brown rat, coproantigens, *Giardia*, Grenada

## Abstract

**Aim:**

*Giardia* is a serious zoonotic parasite, which causes diarrheal disease in humans and animals including rodents. The purpose of this study was to estimate the prevalence of *Giardia* spp. in brown rats (*Rattus norvegicus*) in Grenada.

**Materials and Methods:**

Intestinal contents from 99 and serum samples from 169 brown rats (*R. norvegicus*) from Grenada were collected. These samples were examined for the *Giardia* coproantigens using *Cryptosporidium*/*Giardia* Quik Chek assay (Tech lab^®^ Inc., USA), and the serum was screened through an enzyme-linked immunosorbent assay (ELISA) test kit for *Giardia* antibody (anti-GD) ELISA kit (MyBioSource, San Diego, CA, USA).

**Result:**

*Giardia* coproantigens were positive in 17.17% (95% confidence interval [CI]; 10.33-26.06%) rats, whereas 55% (95% CI: 47.20-62.68) were positive with serum antibodies (anti-GD) to *Giardia*.

**Conclusion:**

The prevalence of *Giardia* spp. in brown rats in Grenada was moderate based on the presence of coproantigens in the intestinal contents and antibody in serum. The findings of Giardia infections and prevalence in brown rats will help veterinarians and physicians to better plan diagnostic and preventative strategies. This is the first report of prevalence of *Giardia* in brown rats in Grenada.

## Introduction

*Giardia* spp. is a flagellate unicellular protozoan causing disease in humans and animals. The disease has a worldwide distribution, but in developing countries, there is a very high prevalence and incidence [1]. Based on the morphology of trophozoite, three species of *Giardia* are recognized: *Giardia lamblia*, synonymous of *Giardia duodenalis*, and *Giardia intestinalis*, is considered one of the major pathogens of humans and animals; *Giardia muris* of rodents, birds, and reptiles and *Giardia agilis* of amphibians [2]. A wide range of animals including rodents is believed to be a reservoir of *G. lamblia* [3,4].

This flagellated protozoan parasite has a direct life cycle. Cysts of *Giardia* spp. are voided in feces from infected host and contaminate food, water, and the environment. Transmission to a new host is through ingestion of food and water contaminated with *Giardia* cysts or direct transmission through ingestion of cysts from person-to-person or animal-to-person contact [5]. This direct transmission usually occurs in unhygienic conditions.

Brown rats (*Rattus norvegicus*) have been found infected with *Giardia* spp. in many countries of the world including Saudi Arabia [6], Poland [7], Turkey [8], and the USA [9]. In Grenada, there has been a report of recent increase in rats and human population, and the likelihood of transmission of zoonotic protozoan infections between these two species has increased as they share common living areas. As far as we are aware, there is no report of *Giardia* spp. infecting rats in Grenada. The objective of the study was to estimate the infection of *Giardia* spp. in brown rats in Grenada.

## Materials and Methods

### Ethical approval

The project entitled “Detection of zoonotic pathogens in brown rats (*R. norvegicus*) in Grenada” was approved by the Institutional Animal Care and Use Committee (IACUC # 16009-R) of the St. George’s University, Grenada.

### Study area

Grenada is the southernmost country in the Caribbean Sea with an area of 348.5 km^2^. The country with low hills, small trees, shrubs and tropical climate is most suitable for brown rats. The country is separated in six parishes: St. Patrick, St. Mark, St Andrew, St. John, St. George, and St. David. Since the trapping of rats was performed near the human dwellings, St. David and St. George parishes, which have more human population compared to other four parishes, were selected for the sample.

### Sample size determination

The sample size was determined using the formula of Glenn [10]. The formula is N=t^2^(p)(1-p)/d^2^, where t=1.96, p=estimated prevalence, and d=desired level of precision. Since prevalence of Giardia for Grenada is not known, we took an average of prevalence from other countries, estimated 10%, and took d=5%. This gave the number of samples 138. Thus, we decided to collect 170 rats, more than estimated number.

### Trapping and collection of rats

One hundred sixty-nine (n=169) rats were collected live from May 1 to July 14, 2017, using traps (45 cm l × 15 cm w × 15 cm h) made manually. Traps were used with cheese or various local fruits as bait. Attempts were made to trap the rats at 10 m radius near the residential buildings. Traps were placed in the evening and visited next day in the morning. Traps with rats were covered with a black cloth and transported to the Necropsy Laboratory of the School of Veterinary Medicine in Grenada. Rats were anesthetized using isoflurane in oxygen through an anesthetic machine (portable vet anesthesia machine isoflurane vaporizer VET CE), manufacturer DRE (Avante Health Solution Company, USA.).

### Collection of samples

The anesthetized rats were weighed and examined physically for their health. Gender was also recorded. Rats below 100 G were grouped as young and over 100 G as an adult following the methodology used by previous researchers [11,12]. Blood was collected from the heart through the thoracic wall and rats were exsanguinated this way. The abdominal cavity of rats was opened using a surgical blade and a pair of forceps. The intestinal tract with contents was placed into containers with 10% formalin until processing.

Sera were separated from the blood by centrifugation at 1500 g for 15 min at room temperature and stored at −80°C till tested.

### Quick check for cyst antigen and enzyme-linked immunosorbent assay (ELISA) for antibody in serum

*Giardia/Cryptosporidium* Quick Chek assay (Tech Lab Inc., USA) was used to detect coproantigens in intestinal contents according to manufacturer’s directions. Serum samples were tested for antibodies against *Giardia* spp. using commercial “qualitative rat *Giardia* antibody (anti-GD) ELISA kit” (MyBioSource, San Diego, CA, USA) following the manufacturer’s recommendations.

### Statistical analysis

The prevalence of *Giardia* spp. was compared by age and gender. The data were analyzed by the statistical methods: Fisher’s exact test, using a GraphPad statistical software (http://www.graphpad.com/quickcalcs/contingency2).

## Results

From 169 tested samples, antibodies for *Giardia* spp. were detected in 93 rats (*R. norvegicus*), with an overall prevalence of 55% (95% confidence interval [CI]: 47.20-62.68%). The result regarding the seroprevalence has been included in Tables-1-3. A significant higher prevalence was found in younger rats (p<0.05). The difference in positive cases between St. George and St. David was statistically not significant (p>0.05).

**Table-1:** Prevalence of *Giardia* antibody (anti-GD) in serum of brown rats from Grenada according to Parish.

Parish positive	Number of sample tested	Number of sample positive	Percentage (%)
St. Georges	76	46	60.5*
St. David	93	47	50.5*
Total	169	93	55.0

* p=0.2159

**Table-2:** Prevalence of *Giardia* antibody (anti-GD) in serum of brown rats from Grenada according to gender.

Parish	Male		Female	
	
	Sample tested	Sample positive (%)	Sample tested	Sample positive (%)
St. Georges	39	24 (61.5)	37	22 (59.5)
St. David	48	22 (45.8)	45	25 (55.6)
Total	87	46 (52.87)*	82	47 (57.3)*

* p=0.6430

**Table-3:** Prevalence of Giardia antibody (anti-GD) in serum of brown rats from Grenada according to age.

Parish	Young		Adult	
	
	Sample tested	Positive sample (%)	Sample tested	Positive sample (%)
St. Georges	12	10 (83.3)	64	36 (56.2)
St. David	7	5 (71.4)	86	42 (48.8)
Total	19	15 (78.9%)*	150	78 (52.0)*

There is a significant association between young and adult rates (p<0.05). P=0.0290

Detection of *Giardia* spp. in intestinal contents was based on the use of antibodies against coproantigens of the organism. The positivity of rats to *Giardia* is presented in Table-4. Of 99 rats tested, 17.17% (95% CI; 10.33-26.06%) were positive for coproantigens in the intestinal content (considering the sensitivity of 98.9% and specificity of 100%).

**Table-4:** Prevalence of coproantigens of Giardia in the intestinal content of brown rats from Grenada.

Parish	Male		Female	
	
	Rats examined	Rats infected (%)	Rats examined	Rats infected (%)
St. Georges	23	4 (17.4)	19	3 (15.8)
St. David	35	6 (17.1)	22	4 (18.2)
Total	58	10 (17.2)^A^	41	7 (17.0)^A^

p=1.00^A^

## Discussion

Conventionally, diagnosis of giardiasis in humans and animals was based on microscopic observation of cysts or trophozoites in feces. New techniques have been developed including ELISA, indirect immunofluorescence and polymerase chain reaction to detect cysts and trophozoites in feces and antibodies in serum to identify past and present infection [5]. In the present study, ELISA was used for the detection of antibodies in serum. Antibody for *Giardia* spp. was found in 55% of the tested rats. Results of seroepidemiology at two sites (St. George and St David) were not significantly different (p>0.05).

Detection of *Giardia* spp. in intestinal contents is based on the use of antibodies to coproantigens of the organism. Of 99 rats tested, 17.17% were positive for coproantigens in the intestinal content. Our result is consistent with previous researchers who also found the similar prevalence of *Giardia* infection in brown rats. Al-Bashan and Sabra [6] reported 19% brown rats infected with *Giardia* in Saudi Arabia, by microscopic examination of feces. Recently, Zeinab *et al*. [13], through microscopic examination, found 19.2% positivity in feces of brown rats in Iran. A lower incidence (6.0%) in feces was found in brown rats and house rats in China [14]. The incidence of *Giardia* in intestine represents current infection, whereas antibody in serum indicates past exposure. In the same set of rats, we found 17.17% rats positive for coproantigens in the intestinal contents compared to 55% rats with antibodies in serum. Martin [9] reported quick clearance of *Giardia* spp. from the intestine as a result of acquired immunity, T-cell playing a role in the process. As a result, the young and immunocompromised animals and humans show delayed clearance of the parasite resulting in severity of the disease.

We found significantly higher prevalence in younger rats. In studies made by previous researchers, prevalence was not affected by sex or age [15,16]. In another study, relation of age with *Giardia* infection was not found [17]. Further studies are suggested to elucidate the relation of age and gender with *Giardia* spp. infection in rats.

## Conclusion

For the first time, the prevalence of *Giardia* spp. was reported in brown rats in Grenada. These rats were considered as reservoir of *Giardia* spp., a serious zoonotic parasite, poses a threat to human population in the country. There is a need to educate the Grenadian community regarding proper maintenance of hygienic conditions in and around their dwellings to prevent survival and proliferation of the rat population. Prevention and control program be implemented by the government and health workers to prevent the contamination of food and water from *Giardia* spp. to combat the zoonosis.

## Authors’ Contributions

RNS: Planning and oversee of the research project and manuscript writing. CCS: Laboratory analysis for coproantigen. AC: Review of the manuscript. JT and ALS: Helping in collection of samples and helping CCS in laboratory work. YS and NR helping KT in ELISA. KT in performing ELISA and analysis of results. All authors read and approved the final manuscript.
